# Developmental Perturbation Induced by Maternal Asthma during Pregnancy: The Short- and Long-Term Impacts on Offspring

**DOI:** 10.1155/2012/741613

**Published:** 2012-07-08

**Authors:** Vicki L. Clifton, Michael Davies, Vivienne Moore, Ian M. R. Wright, Zainab Ali, Nicolette A. Hodyl

**Affiliations:** ^1^Robinson Institute, University of Adelaide, Adelaide, SA 5005, Australia; ^2^Robinson Institute, University of Adelaide, Lyell McEwin Hospital, Haydown road, Elizabeth Vale, SA 5112, Australia; ^3^Discipline of Public Health, University of Adelaide, Adelaide, SA 5005, Australia; ^4^Mothers and Babies Research Centre, Hunter Medical Research Institute, University of Newcastle, NSW 2310, Australia

## Abstract

Maternal asthma is a common disease to complicate human pregnancy. Epidemiological studies have identified that asthma during pregnancy increases the risk of a number of poor outcomes for the neonate including growth restriction, lower birthweight, preterm delivery, neonatal resuscitation, and stillbirth. Asthma therefore represents a significant health burden to society and could have an impact on the lifelong health of the children of women with asthma. Our research has identified that maternal asthma in pregnancy induces placental dysfunction and developmental perturbation in the fetus in a sex specific manner. These alterations in development could increase the risk of metabolic disease in adulthood of children of asthmatic mothers, especially females. In this paper, we will discuss the evidence currently available that supports the hypothesis that children of mothers with asthma may be at risk of lifelong health complications which include diabetes and hypertension.

## 1. Introduction

Asthma is a noncommunicable chronic disease that has major health, social and economic burdens in society. Asthma is generally viewed as a chronic inflammatory condition of the lung associated with airway remodelling, reversible airway obstruction, and bronchospasm accompanied by frequent symptoms of wheeze, cough, and shortness of breath. It occurs in genetically susceptible individuals and is often initiated by exposure to allergens, exercise, or viral infection. The mechanisms leading to its symptoms can be diverse suggesting there are numerous phenotypes of this chronic disease [1]. The major questions related to chronic disease are how to prevent them, effectively treat disease states, and minimise handicap. Our research work focuses on both primary prevention for the next generation and management of conditions in the current generation of women. This paper considers opportunities for clinically improving fetal outcomes in pregnancies complicated by asthma, particularly with regard to reducing the risk of metabolic disease in offspring. 

Asthma is the most prevalent chronic disease to complicate pregnancy in Australia, affecting 12% of pregnant women and could increase to 20% in the next 5 years [2]. Epidemiological studies we conducted in New South Wales, Australia have identified that maternal asthma during pregnancy increases the risk of a number of poor outcomes for the neonate including growth restriction, lower birthweight, preterm delivery, neonatal resuscitation, and stillbirth [3]. These findings are in agreement with international reports on outcomes of pregnancies complicated by asthma [4, 5]. Asthma therefore represents a significant health burden to society and that is expected to have an impact on the lifelong health of the children of women with asthma.

The *in utero* environment can induce plastic changes in the fetus and infant predisposing them to disease in later life [6]. Specifically a lower birth weight has been shown to be associated with an increased risk of cardiovascular and metabolic disease [6]. Furthermore perturbations in fetal development are not just limited to intrauterine growth restriction (IUGR) but associated with birth weights within normal ranges [7]. Studies in laboratory animals have identified the underlying mechanisms associated with disease risk in adulthood and have shown that offspring at particular risk are those animals who had restricted growth *in utero* followed by accelerated compensatory growth of certain body compartments, particularly fat mass before maturity [8]. Studies in humans show that the association between birthweight and later disease development is independent of socioeconomic status and contributions of known adult lifestyle factors that increase disease risk [7]. Together these findings have contributed to the predictive adaptive response (PAR) hypothesis [9] where a developing organism predicts the external postnatal environment based on the *in utero* conditions and subsequently alters its development to optimise survival in the predicted environment. This survival strategy increases the chance of survival to reproductive age at the risk of health consequences in later life. 

The *in utero* mechanisms underlying developmental perturbation are broadly categorised into four areas: (1) epigenetics, (2) mitochondrial function, (3) organ and tissue development, and (4) homeostasis control systems [7]. Maternal disease during pregnancy could lead the fetus to inappropriately adjust its development through these four mechanisms which then leads to a mismatch between the individual and its external environment. We have evidence to suggest that maternal asthma during pregnancy is a disease that contributes to placental dysfunction and developmental perturbation in the fetus [10]. Based on our findings [3] and a large body of work conducted by others [11], we expect that maternal asthma has an intergenerational impact and increases disease risk in the offspring [10, 12]. Moreover, these perturbations can be sex specific whereby females reduce their size in response to the presence of maternal asthma though they are not growth restricted (<10th birthweight centile) [13]. Male fetuses appear to continue to grow normally in response to maternal asthma [13]. However if asthma worsens with an exacerbation then the female fetus continues to be small but not growth restricted while the male responds adversely being either growth restricted or delivered preterm or stillborn [14]. These specific alterations in development could increase the risk of metabolic disease in adulthood of children of asthmatic mothers through the mechanisms we outline below [13, 15–22].

## 2. Increased Placental Vascular Resistance in Pregnancies Complicated by Asthma

Animal studies have demonstrated that the placenta is important in regulating the development of the fetal cardiovascular system. The placental vascular bed remains in a predominantly vasodilated state throughout gestation. The fetal side of the circulation is directly supplied by fetal cardiac output that is maintained and constant relative to size for most of the last half of gestation. When there is increased placental vascular resistance, the fetal heart adjusts its cardiac output through changes in its growth to maintain a constant stroke volume and supply to the placenta. In the sheep model of increased placental vascular resistance there were significant effects on the fetus including growth restriction, myocardial hypertrophy, hypoxaemia and hypertension [23]. This study provides evidence that altered placental vascular function and morphology can have an adverse effect on fetal cardiovascular development. 

Our research has shown that placental vascular structure and function [15] are significantly altered in the presence of maternal asthma in a sex-specific manner. In the presence of maternal asthma placental structure was altered in the placentae of male fetuses as demonstrated by a significant decrease in capillary volume [16]. Other structural changes in the placentae of male fetuses of pregnancies complicated by asthma where mothers' were treated with high dose inhaled corticosteroids included decreased capillary length and villi length [16]. Placental vascular function studies identified that both male and female placentae from asthmatic pregnancies had reduced vasodilatory capacity suggesting the presence of increased vascular resistance [15]. Increased placental vascular resistance may have an impact on *in utero* cardiovascular development in children of asthmatic mothers.

## 3. Altered Homeostatic Control in Pregnancies Complicated by Asthma

Homeostatic control may be one mechanism that contributes to developmental perturbation through altering the set points for hormonal balance and changing tissue sensitivity to many hormones. Prenatal exposure to excess maternal glucocorticoids is one example of re-setting a homeostatic control set-point. In the placenta, 11*β*-hydroxysteroid dehydrogenase (11*β*-HSD2) protects the fetus from the potentially harmful effects of endogenous maternal glucocorticoids, by acting as a barrier from the much higher levels of cortisol found in the mother [24]. The main functional isozyme in the placental syncytiotrophoblast is 11*β*-HSD2 converting cortisol to cortisone. Excess glucocorticoids have potentially adverse effects on the fetus and have been linked to altered fetal programming which includes decreased growth [25, 26], reduced kidney nephron number [27], and reprogramming of the fetal hypothalamic-pituitary adrenal axis [28]. Several studies have described a relationship between reduced activity of 11*β*-HSD2 and reduced birth weight or intrauterine growth restriction [29, 30]. 

Excess exposure to glucocorticoids at a critical time point in kidney development has been shown to reduce nephron number and predispose an individual to hypertension in later life [27]. Tang et al. [31] have reported that prenatal exposure to excess glucocorticoids in the rat model was associated with salt-sensitive hypertension in adult offspring due to decreased renal 11*β*-HSD2. In humans, placental 11*β*-HSD2 activity and cortisol exposure *in utero* were associated with blood pressure at age 3 years [32]. Each unit increase in cortisol exposure was associated with 1.6 mmHg increase in blood pressure [32]. Human and animal females are more susceptible to increased cortisol exposure in utero and subsequently develop hypertension in adulthood due to elevation of the renin-angiotensin system [33].

Our prospective study of pregnant women with asthma has shown that asthma alone and not the inhaled steroids used to treat maternal asthma has the greatest effect on the fetus. We have found that asthmatic women who do not use inhaled steroids for treatment have smaller female neonates at term [13]. This late gestation reduction in fetal growth was associated with a significant decrease in placental 11*β*-HSD2 activity and a significant decrease in female fetal adrenal status [13] indicating female babies of asthmatic mothers are prenatally exposed to higher concentrations of glucocorticoid which may predispose them to cardiovascular disease in later life. If mothers' used inhaled glucocorticoids to treat their asthma during pregnancy there was a significant increase in placental 11*β*-HSD2 activity in placentae of females. We have also recently identified that inhaled steroids do not cross the placenta and affect fetal adrenal status during pregnancy [19] suggesting that only changes in endogenous maternal cortisol concentrations and adjustments in placental 11*β*-HSD2 activity influence fetal growth in pregnancies complicated by asthma. We have recently reported similar sex specific adjustments in of placental 11*β*-HSD2 activity in preterm placentae following exposure to betamethasone. Placental 11*β*-HSD2 activity was increased in female placentae and adrenal status altered but there were no changes in male placentae [34]. These studies suggest that in pathological pregnancies the female placenta adjusts 11*β*-HSD2 activity in response to the maternal glucocorticoid environment. However, there are no corresponding data to indicate that the male placenta has the capacity to adjust placental 11*β*-HSD2 activity in response to the maternal environment. This may be one mechanism that confers a survival advantage in the female fetus relative to the male fetus but increases the risk of cardiovascular disease in later life in females relative to males.

## 4. Differential Mitochondrial Gene Expression in Pregnancies Complicated by Asthma

Pathophysiological mechanisms that contribute to the development of hypertension and diabetes include impaired mitochondrial oxidative phosphorylation and mitochondrial biogenesis which lead to myocardial and endothelial dysfunction [35] and insulin resistance and pancreatic beta cell failure [36], respectively. Recent placental studies report that several mitochondrial genes and proteins are altered in pregnancy complications such as maternal obesity [37], treatment of maternal HIV infection [38], maternal undernutrition [39], and hypoxia [40]. Microarray studies of the placenta have identified a number of mitochondrial genes are altered in the presence of maternal asthma during pregnancy in both males and females [22] including ATP synthase 2B4 (ATP2B4), cytochrome p450 (CYP19A1), catechol-O-methyltransferase (COMT), coproporphyrinogen oxidase (CPOX), and malate dehydrogenase 2 (MDH2). Glyceraldehyde 3-phosphate dehydrogenase (GAPDH) and MDH2 are enzymes catalyzing reactions in glycolysis and the citric acid cycle in the mitochondrial metabolic pathway). CPOX has an important role in heme (Fe) production which is vital for oxygen transport in the blood. COMT is an enzyme important for catecholamine metabolism and catabolism such as for dopamine, noradrenalin, and adrenaline. Some of these genes were altered sex specifically in the placenta of asthmatic women, including GAPDH which was only altered in males, while MDH2, COMT, and CPOX were decreased in female placentae of pregnancies complicated by asthma. The presence and absence of inhaled glucocorticoid use for asthma treatment during pregnancy also influenced mitochondrial gene expression, with MDH2, COMT, and CPOX gene expression being increased in placentae of females whose mothers' used these inhaled steroids during pregnancy. Since it is proposed that programming of mitochondria is the key adaptation enabling the fetus to survive in a poor in utero environment [41, 42], alterations in the expression of each of these genes may contribute to developmental perturbation in pregnancies complicated by asthma but this requires further examination. 

## 5. Sex Specific Placental Gene Expression and Protein Function and Its Relationship to Fetal Growth in Pregnancies Complicated by Asthma

Numerous gene changes in placentae of male and female fetuses of pregnancies complicated by asthma have been reported [12, 17, 19, 21]. However most of the gene changes have been observed specifically in the presence of a female fetus, accompanied by a lower birthweight for this sex. Global gene changes assessed using microarray identified sex specific effects, such that female placentae of pregnancies complicated by asthma had 59 gene changes compared to 6 gene changes in the placentae of males [22]. The main gene networks altered in the female placentae were genes associated with inflammation, cellular growth, and proliferation suggesting these are the pathways that contribute to reduced growth in females [22]. The differences in inflammatory gene expression have also been confirmed showing that female placentae of pregnancies complicated by asthma have increased expression of a range of proinflammatory genes relative to male placentae [17]. Conversely the male fetus continues to grow normally in the presence of chronic, maternal asthma [13] due to an increase in the production of IGF-1 [43] and a limited alteration in global gene expression [22]. 

Responsiveness to glucocorticoids and the sensitivity of the glucocorticoid receptor appear to be central to the differences in developmental perturbation in pregnancies complicated by asthma. Female placental function and growth appears dependent on exposure to cortisol whereas males appear glucocorticoid resistant, with the consequence that cytokine gene expression is dependent on cord blood cortisol concentrations in females but not in males [17]. We have reported that female birthweight is negatively correlated with cord blood cortisol but no such association exists in males [44]. We initially proposed that these differences in the response to cortisol were due to sex specific differences in placental GR*α*, the bioactive isoform of the glucocorticoid receptor. However in both whole placental tissue [44] and *in vitro* studies [18], GR*α* protein expression did not vary with asthma or between the sexes. This difference may be conferred by a difference in placental 11*β*-HSD2 activity as we have already discussed, but it may also be related to differences in GR*α* protein function. An alternative explanation is that cytokine gene expression is dependent on cord blood cortisol concentrations in females but not in males [17]. It has been observed in *in vitro* work with placental explants that female placentae rapidly inhibited placental cytokine production in the presence of cortisol but this was not observed in male placentae, even though receptor protein levels were the same [18]. There are several isoforms of the glucocorticoid receptor expressed in the placenta [45] that may interact with GR*α* to exert sex specific differences in glucocorticoid sensitivity and this is the subject of current investigations in our laboratory.

Several studies have shown that epigenetic alterations in GR may contribute to differences in GR protein expression [46]. Maternal stress during pregnancy was identified to contribute to increased methylation of the GR gene and associated with increased salivary cortisol concentrations in neonates at 3 months [47]. Dietary protein restriction during pregnancy in the rat contributed to epigenetic modification of GR and increased expression of the GR protein in the liver of offspring [48]. These studies provide evidence that epigenetic modification of GR may influence protein expression levels, however, there is no evidence at present to suggest that placental GR is epigenetically modified in the presence of maternal asthma because GR protein concentrations do not vary between groups or sex. Therefore we continue to focus on post translational modification and GR protein function. 

Sex specific differences in developmental perturbation due to differences in placental gene expression and protein function in pregnancies complicated by asthma are associated with developmental perturbation and may contribute to disease risk in later life. Preliminary evidence in relation to childhood growth would support this hypothesis.

## 6. Childhood Growth in Pregnancies Complicated by Asthma

**Figure 1:**
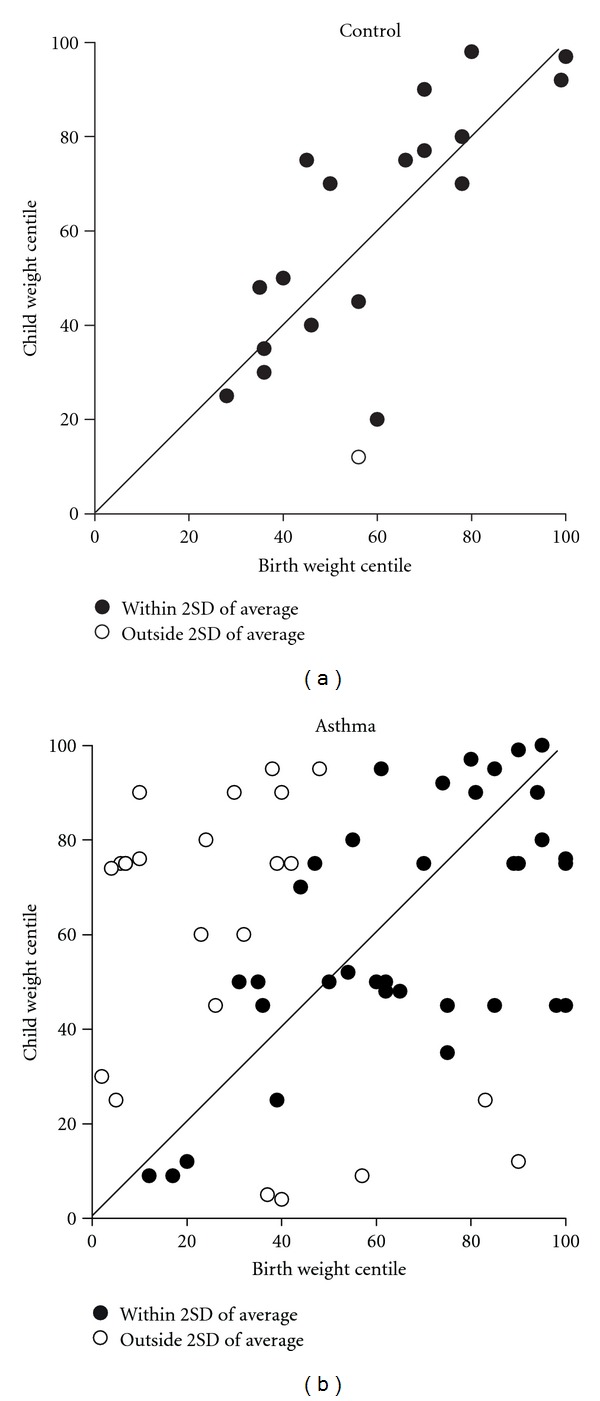
The association between birth weight centile and child weight centile (measured at 18 months) in children of healthy mothers (a) and mothers with asthma during pregnancy (b). Data points represent those children whose growth falls within two standard deviations (solid circles) of the mean growth trajectory, as indicated by the solid line, and those that fall outside of 2 standard deviations (open circles).

We have followed a cohort of pregnant women (*n* = 694) in Newcastle from 1999 to 2007 and plan to follow up the children who currently range from the ages 6–13 years. A great deal of information relating to maternal asthma severity, asthma treatment used during pregnancy, delivery details, and neonatal outcomes were collected from this cohort, and numerous publications have been generated on maternal health during pregnancy [14, 49–52], placental function [17, 18, 22], and fetal growth [43]. Our preliminary investigations of the children in this cohort (conducted at age 18 months) demonstrated altered growth trajectories in children whose mothers had asthma during pregnancy. These data show that while children of nonasthmatic mothers did not deviate from their birth growth trajectory, as projected from their birth weight centile, 40% of children of asthmatic mothers had already deviated from this trajectory at 18 months of age (Figure 1), with the majority (78%) demonstrating accelerated growth also termed as “catch up growth.” Interestingly there was a sex difference in the children that were 2 standard deviations away from the predicted growth trajectory with 60% of females displaying accelerated growth relative to 40% of males. Children in this preliminary study were not significantly different in relation to maternal and neonatal characteristics including maternal age, BMI, maternal cigarette use, gravidity, parity, birthweight, birthweight centile, or placental weight. This group was a representative sample of the entire cohort.

## 7. Accelerated Growth and Metabolic Syndrome

Human and animal studies have provided evidence that low birthweight followed by rapid postnatal weight gain are associated with adult obesity, diabetes, cardiovascular disease and premature mortality [8]. Most of the accelerated weight gain is in the form of adipose tissue rather than lean tissue with numerous studies reporting deposition predominantly in the abdomen though usually total body fat is increased overall [8]. Studies from the UK, Denmark, and Finland report that adults who were small for gestational age still had a greater abdominal fat content relative to adults of similar height, weight, age, and sex [8]. Increased abdominal fat predisposes individuals to an increased risk of hypertension and diabetes with aging. The presence of abdominal fat increases sympathetic nervous system activity stimulating activation of the kidney renin-angiotensin system and causing peripheral vasoconstriction and hypertension [53]. Other mechanisms that are altered *in utero *and may contribute to the development of hypertension are microvascular structure and function [54] and altered hypothalamic-pituitary adrenal function [55]. The *in utero* mechanisms that contribute to subsequent development of type 2 diabetes are proposed to be epigenetic, specifically related to the histone acetylation and subsequent suppression of Pdx1, a transcription factor that is involved in the development of the pancreas especially in beta cell development [56]. The later development of insulin resistance and type 2 diabetes is a combination of increased adiposity leading to increased lipolysis and circulating fatty acids, inflammation, mitochondrial dysfunction and aberrant insulin signaling [57]. The exact order of later life events predisposing to diabetes appears uncertain. However accelerated growth and adiposity are currently the strongest indicators of risk.

## 8. Followup Studies of Children from Pregnancies Complicated by Asthma

The neonatal outcomes of pregnancies complicated by asthma are now well characterised including birthweight, hospitalisations, and birth defects. The long-term outcomes are limited with only two studies that examined the development of children of asthmatic mothers. Gordon et al. [58] reported that there was a significant increase of neurological abnormalities at 1 year of age in children of asthmatic mothers with poorly managed asthma. Shatz et al. reported no differences between children, at 15 months of age, of asthmatic women who had well-controlled asthma during pregnancy and non asthmatic women [59]. Both of these studies did not follow up the children beyond 2 years of age. It would be important to also determine whether there are any long term effects of asthma and its treatment on childhood development and adult disease risk. Very few studies have examined children long term to determine when early pathophysiological changes that result from developmental perturbation are discernable and there is no evidence in pregnancies complicated by asthma. 

## 9. Future Studies

Aside from the followup of cohorts of children who were exposed to maternal asthma during pregnancy to determine their susceptibility to disease in later life, there are several unanswered questions in this field. The exact *in utero* and early life mechanisms that alter immune, metabolic and cardiovascular development of offspring of mothers with asthma and the identification of biological pathways that could be targeted for preventative interventions are yet to be described. Some of these areas may be addressed in appropriate animal models of asthma which are currently limited to several murine models [60] and a sheep model [61] of allergen induced asthma. These models could be adapted for pregnancy related studies and offspring followup, and used for the elucidation of mechanisms that contribute to altered health outcomes as well as to test pregnancy-related or early life interventions. Animal models of asthma may also be used to discern the impact of asthma on child health outcomes separately from the impact of asthma treatment effects, which cannot be done in humans for ethical reasons. This is because it is inappropriate to randomise asthmatic women to a no asthma treatment regime in order to determine the effect of asthma alone. 

## 10. Clinical Implications

The current research of the effect of asthma during pregnancy on fetal growth and development has identified that chronic asthma, regardless of its severity, and acute asthma exacerbations contribute to the poorest outcomes for the fetus. The use of inhaled corticosteroids to control asthma during pregnancy protects the fetus from the inflammatory effects of maternal asthma and reduces the incidence of exacerbations which in turn reduces the risk of a poor outcome for the fetus. This work suggests asthma control, and appropriate use of inhaled steroids is an important intervention in pregnancy. However the long-term effects of asthma and its treatment on the fetus are yet to be defined. 

## 11. Conclusion

In combination, these studies suggest that the presence of maternal asthma can impact on fetal growth, neonatal health, and development. The perturbations observed in placental function and fetal growth suggest the fetus institutes a predictive adaptive response to maternal asthma during pregnancy. The impact of maternal asthma on fetal development is sex specific and suggests the female fetus may be more at risk of metabolic disease as indicated by greater exposure and sensitivity to changes in glucocorticoid concentration and accelerated growth in early life relative to the male. Information from animal studies would suggest that adult health is significantly affected by these perturbations. However further investigations in human cohorts are required, and current maternal asthma and pregnancy cohorts will provide further evidence as children and adult offspring are studied in the future.
